# Initial Experience of a Cohort of Patients With Hypertrophic Cardiomyopathy Undergoing Biventricular Pacing

**Published:** 2011-02-08

**Authors:** Christopher A Rinaldi, Senthil Kirubakaran, Clifford A Bucknall, Julian Bostock, Jaswinder S Gill

**Affiliations:** Cardiothoracic Dept Guys and St Thomas NHS Trust

**Keywords:** pacemakers, hypertrophic cardiomyopathy, conduction, biventricular

## Abstract

**Background:**

Dual chamber pacing improves functional status and reduces left ventricular outflow tract gradients in some, but not all patients with hypertrophic cardiomyopathy (HCM) by altering ventricular depolarisation. We investigated the use of biventricular (BIV) pacing in symptomatic patients with HCM.

**Method:**

8 patients aged 58±7yrs with symptomatic HCM underwent BIV pacing. 5 patients had LVOT gradients >30mmHg. Ventricular electrodes were placed in the right ventricle (RV) and a branch of the coronary sinus. An atrial electrode was inserted to achieve BIV pacing with a short AV delay. The short-term effects of different pacing modalities were assessed using 2-D and Doppler echocardiography. Symptoms and exercise tolerance were assessed after a month of each pacing mode. Long-term follow up data was available for 5 years.

**Results:**

Baseline EF was 67±14% and mean QRS duration was 132±26msecs. BIV pacing reduced QRS duration compared to RV pacing (129±46 vs. 205±54msecs, p<0.005). Five of the seven patients had baseline LVOT gradients (mean 67±25mmHg) that decreased to 41±15mm Hg with RV pacing (p<0.01) and 25±15mmHg with BIV pacing (p<0.005). Improvements in exercise time with active pacing occurred in six out of eight patients (75%), three (37.5%) had optimal exercise times with RV pacing and three with BIV pacing. Of the three patients with short term improvements with BIV pacing, one died 4 years post implant, one deteriorated with LV dilatation and one had the system explanted for infection.

**Conclusion:**

BIV pacing showed short-term beneficial effects in some patients over and above RV pacing alone.

## Introduction

The mainstay of treatment for symptomatic patients with hypertrophic cardiomyopathy (HCM) is medical therapy, with surgical resection or alcohol septal ablation performed in patients with refractory symptoms and a significant left ventricular outflow tract (LVOT) gradient. Initial observational and uncontrolled studies reported benefits with dual chamber pacing with regard to symptoms and LVOT gradients [1-3]. This was thought in part to be due to altered LV activation from RV apical pacing causing an increase in LV systolic diameter and reduced systolic anterior motion of the mitral valve and therefore reduced LVOT gradients. Later however, randomized cross-over trials reported less beneficial effects in terms of exercise capacity and LVOT gradient reductions [4-6]. There are in addition a number of patients who do not derive any benefit from dual chamber pacing, and as a result this treatment modality is reserved for severely symptomatic and medically refractory HCM patients with LV outflow obstruction [7]. We reported the first case of the beneficial effects of biventricular pacing in this patient population in terms of symptomatic status, exercise times and LV outflow tract gadients [8]. We hypothesized that some patients with HCM have evidence of intraventricular conduction delay which is increased by RV pacing and therefore could benefit from biventricular pacing. We therefore looked to examine its role in a cohort of highly symptomatic patients over prolonged follow up.

## Methods

### Patients

The local research ethics committee approved the protocol and all patients gave written informed consent. Table 1 shows patient characteristics. All had symptomatic HCM despite medical therapy. Three patients had existing dual chamber pacemakers, 2 had undergone alcohol septal ablation and one a surgical myectomy. 7 patients (88%) had a QRS duration >120msecs and 5 patients had LVOT gradients >30mm Hg at rest.

### Implantation of pacemaker

Implants were performed over the period from January 2001 to January 2002.  In 3 patients an existing DDD pacing system was upgraded. An atrial electrode was positioned in the right atrial appendage and a right ventricular pacing lead (if not already in situ) to the RV apex. A dedicated unipolar left heart pacing lead [Uni Aescula LV model 1055K-(Pacesetter-St Jude)] was placed to a branch of the coronary sinus from which adequate pacing and sensing of the LV could be performed. The RV lead was positioned as far as possible from the LV lead.  A DDDR pulse generator capable of simultaneously pacing both right and left ventricles was implanted in the left pectoral region. [Chorum 7336B DDDR (ELA Medical), Montrouge France, MSP in 7 patients, Medtronic In Sync III Medtronic, Minneapolis, USA in 1 patient].

### Programming

Pacemakers were randomly programmed to either no active pacing (A) RV DDD pacing (B) or biventricular DDD pacing (C) in a serial crossover fashion. Patients were paced in modalities B and C at a rate of 70 beats/min with a short AV delay of 78 msecs for maximal ventricular pre-excitation. Patients were blinded to the pacing mode in an attempt to minimise placebo effect.

### Main outcome measures

#### Evaluation of patients

Patients were evaluated in the short term after 4 weeks in each pacing modality. Objective exercise tolerance was assessed with [16] treadmill exercise testing from which responders were defined as patients who had an improvement in exercise capacity. Symptomatic status was assessed using the Minnesota Living with Heart Failure Questionnaire [16]. 2-D echocardiography was performed using a series 5 Vingmed (Vingmed Inc) to assess cardiac chamber dimensions and septal thickness. LVOT gradients were measured using Continuous Wave Doppler echocardiography. Patients that responded to BIV pacing were subsequently left in that modality and long-term follow up was assessed after 5 years of treatment.

### Statistical analysis

Data are expressed as mean ±SD. Pacing parameters were analysed using ANOVA and post Hoc with Scheffes f test and a p value of < 0.05 was considered significant.  Statistical analysis was performed on an Apple Macintosh iBook Computer (Apple Computers Inc., Cupertino, CA) using Statview 512+™ (Abacus Concepts, Berkeley, CA)

## Results

A BiV pacemaker was successfully implanted in 8 out of 9 patients.

### Baseline characteristics (Table 1)

The mean age was 57±8 years and 75% were male. The mean QRS duration was 132±26ms, LVOT gradient 67±25 mmHg, ejection fraction (EF) 67±14% and average interventricular septal thickness was 2.0±0.6cm.

### Short term end points  (see Table 2)

Six patients (75%) had objective improvements in exercise capacity with RV or BIV pacing (responders) and two had no benefit or a reduced exercise capacity (non responders). Of the responders, 3 had better exercise capacity with BIV pacing (Group 1) and 3 with RV pacing (Group 2). Those that responded to BIV pacing compared to RV pacing tended to be more symptomatic (symptoms scores: BIV 79±6, RV 55±45), have have reduced exercise times (BIV 113±102 secs, RV 352±20), larger LVOT gradients (BIV 81±19, RV 45±12 mmHg) and a longer QRS duration (BIV 145±20, RV 120±36ms) at baseline. Of the non responders, one patient had no objective improvement in exercise tolerance with either pacing modality despite a symptomatic improvement. Another had worsening of exercise tolerance with pacing despite a symptomatic improvement. This would suggest a significant placebo effect in these two patients.

### Responders vs Non responders

There was no significant difference between responders and non responders in terms baseline QRS duration (133±30ms, 131±15ms respectively) or exercise times (232±164ms, 219±200), however there was a significant difference in baseline LVOT gradient (51mmHg, 25mmHg respectively).

### Group 1 (BIV responders, n=3)

3 patients had maximal improvements in exercise time AND symptoms with BIV pacing.

**Figure 1:**
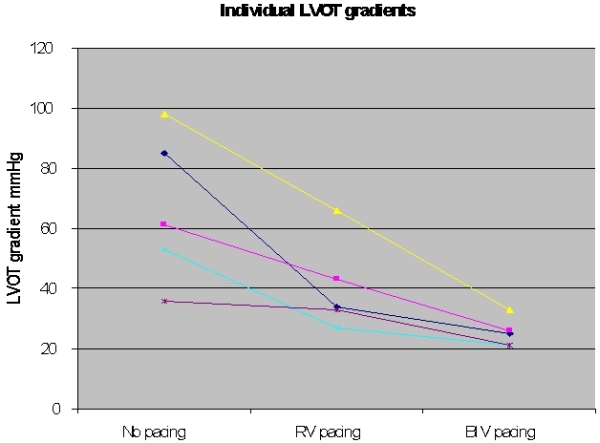
Changes in LV outflow tract gradient in each patient

Baseline QRS was 145±20msecs, increasing to 211±50ms (p<0.05) with RV pacing and decreased to 133±50ms with BIV pacing. (p=ns) Baseline exercise time was 113±102 secs which was unchanged with RV pacing (105±65 secs) but increased to 205 ±137 secs with BIV pacing (p<0.05 vs. no and RV pacing). Symptoms scores progressively reduced (implying less symptoms) from 79±6 with no pacing to 69±15 with RV pacing to 35±16 with BIV pacing (p<0.05). Similarly there was a progressive fall in the LVOT gradients from 81±19mm Hg with no pacing to 48±17 with RV pacing and to 28±4 with BIV pacing (Figure 1).

### Group 2 (RV pacing optimal)

3 patients had better exercise tolerance with RV pacing compared to baseline and BIV pacing. Baseline QRS with no pacing was 120±36 msecs, 200±96 msecs with RV pacing and 121±33msecs with BIV pacing. Baseline exercise time was 352±120 secs increasing to 516±136 secs with RV pacing (p<0.05) and was 481±113 secs with BiV pacing. Despite better exercise times in this group with RV pacing, BIV pacing was associated with a further reduction in symptoms scores (55±45 at baseline, 36±39 with RV pacing and 26±35 with BiV pacing) and LVOT gradient (45±12mmHg at baseline decreasing to 30±4mmHg with RV pacing and 21±4mmHg with BIV pacing) when compared with RV pacing.

### Long term follow up (see Table 3)

Patients were left in the pacing mode, which showed greatest short-term benefit in terms of exercise times. Follow up is available on all patients 5 years since device implantation.

#### Group 1

Of the three patients that showed a short-term benefit with BIV pacing one patient died 43 months after their implant due to a gradual deterioration in LV function with end stage heart failure. One patient has deteriorated clinically and had entered the dilated phase of HCM. The third patient after an initial improvement deteriorated with development of atrial arrhythmias and needed explantation of the system due to pacemaker system infection and currently has a single chamber pacemaker.

#### Group 2

All 3 patients that were responders to RV pacing remained well. Two have had their pacemakers upgraded to ICDs. One at 36 months for non-sustained VT and one at 82 months post implant due to syncope, non-sustained ventricular tachycardia (NSVT) and a positive family history for sudden cardiac death.

#### Non-responders shortly after LV lead implantation

Of the non-responders, one patient developed NSVT with syncope. At that time is was not possible to individually switch off the LV lead, therefore the lead was extracted and upgraded to a dual chamber ICD 3 months following implantation. No further VT was documented following this during the 5 year follow up. The other patient died of progressive heart failure 36 months post implant.

## Discussion

This is the first case series looking at the short term and long term effects of biventricular pacing in symptomatic patients with hypertrophic cardiomyopathy. In this small series there appeared to be a beneficial short term effect with pacing on exercise times, symptoms and outflow tract gradients in some but not all highly symptomatic patients. Of those with objective short term benefit, 50% appeared to have an incremental benefit from BIV pacing associated with a dramatic reduction in left ventricular outflow tract gradients. The mechanism of benefit from biventricular pacing in this small group of patients is unclear. Part of its beneficial effect could be related to alteration of the left ventricular contraction pattern. Simultaneous activation of the right and left posterolateral wall (the site of the LV pacing lead) would cause earlier apical contraction and later activation of the basal septum which could result in a decrease in the LVOT gradient. Those benefiting from BIV pacing had more severe exercise limitation, worse symptoms and larger outflow tract gradients. BIV pacing appeared to have an incremental effect on LVOT gradients over RV pacing alone. In one patient implanted with a device capable of independently altering VV timings it was possible to alter the gradient depending on the relative timing of LV and RV activation with greatest gradient reduction when LV was paced 4 msecs prior to the RV, with earlier LV activation times being not as effective in reducing the gradient. Of the patients with short term improvements from BIV pacing, two developed progressive LV dysfunction. This may simply represent the natural history in these patients with the severe forms of HCM degenerating into the dilated phase of their disease, rather than an effect of BIV pacing.

Other points of note are the fact that several of the patients subsequently had ICDs and in one patient there was the suggestion that LV pacing may have been arrhythmogenic with non-sustained VT developing, which improved after LV lead extraction.

### RV pacing and hypertrophic cardiomyopathy - previous studies

Dual chamber RV pacing was initially proposed as an alternative therapy for symptomatic patients with hypertrophic cardiomyopathy. Its beneficial effects were thought to be explained by alteration of LV depolarization and optimization of atrial contraction [9,10]. Initial uncontrolled studies suggested significant beneficial effects of dual chamber pacing on LVOT gradients and symptomatic status [11]. However later randomised studies produced conflicting results. Kappenberger et al [5,12], in a study of 83 patients showed improved symptoms, exercise duration and LVOT gradients with RV pacing. Nishimura et al, in a double blind randomised crossover trial recruited 21 patients with severe symptoms of HCM and showed that dual chamber (RA and RV) pacing relieved symptoms and decreased gradients in some patients however in others symptoms didn't change or worsened [6]. In addition it was noted that chronic DDD pacing while reducing obstruction may exacerbate diastolic dysfunction is these patients [13,14]. Symptomatic improvements also occurred from implantation of the pacemaker without its haemodynamic benefit, suggesting a significant placebo effect. Maron et al, studied the effect of dual chamber pacing in 48 symptomatic HCM patients all with LVOT gradients greater than 50 mm Hg at rest [15]. Six months of unblinded pacing in these patients resulted in functional class and quality of life improvements but no change in peak oxygen consumption. The authors concluded that symptomatic improvement was more consistent with a placebo effect. Modest reductions in outflow gradient were achieved in most patients with a small subset of patients over 65 years of age showing a clinical response, suggesting that DDD pacing could be a therapeutic option for some elderly patients.

### BIV pacing and hypertrophic cardiomyopathy

Biventricular pacing has been shown to be beneficial in symptomatic patients with heart failure, with a number of studies demonstrating improved morbidity, mortality and its effects on reverse remodeling [16-30]. In view of these results, case studies were reported demonstrating the beneficial effect of biventricular pacing in end-stage hypertrophic cardiomyopathy (severely impaired left ventricular function). Pezzulich et al, [31] reported a case of a 43 year old with severely impaired LV systolic function and hypertrophic cardiomyopathy. Following biventricular pacing there was a significant improvement in clinical status, left ventricular ejection fraction and peak VO2. A similar case report was later reported in a 42 year old patient with dilated-hypokinetic hypertrophic cardiomyopathy [32]. Following this a study of twenty patients with non-obstructive HCM and symptoms of heart failure and effects of biventricular pacing was reported by Elliot et al [33]. After a mean follow up of 13 months, improvement in symptoms was reported in 40% of patients. This was associated with an increase in ejection fraction, a reduction in left ventricular end-diastolic diameter and left atrial diameter. There was however no change in peak oxygen consumption during exercise.

We reported the first case of the beneficial effect of biventricular pacing in a symptomatic patient with hypertrophic cardiomyopathy, preserved LV systolic function and a significant LVOT gradient [8]. We found a significant improvement in symptomatic status, exercise times and haemodynamics over and above that produced by RV DDD pacing and far superior to no pacing. Following this three case reports were published demonstrating its beneficial effect on symptomatic patients with HCM in children and adults [34-36].

## Limitations

This is a small series of patients and thus it is difficult to generalise the results. The patients who appeared to show a short term benefit with BIV pacing were a highly symptomatic group with significant electrical dyssynchrony. There also appeared to be a placebo effect in some patients who were paced. No measure of mechanical dyssynchrony were made with tools such as Doppler tissue imaging and therefore it is unclear as to whether improvements with BIV pacing were related to improved mechanical synchronisation. Also as the majority of devices were unable to program independent VV timing it is unclear as to whether optimisation of such timings with echocardiography may have been able to provide further benefit.

## Conclusion

We have shown short-term objective improvements in a small sub group of patients with HCM with the use of BIV pacing. With longer term follow up this benefit does not appear to be sustained. These findings require further investigation in a larger patient population in a randomised manner in the current era of devices with independent V-V programming to ascertain a potential mechanism of benefit.

Biventricular pacing could therefore become an attractive alternative for alcohol septal ablation or surgical ablation in symptomatic patient with hypertrophic cardiomyopathy, especially in subjects requiring an ICD.

## Figures and Tables

**Table 1:**
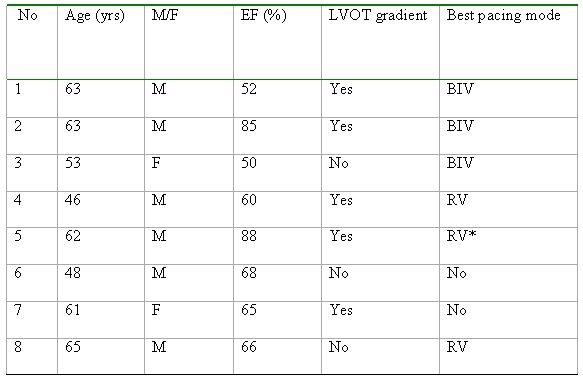
Baseline patient characteristics

* Symptomatically better with BIV pacing but greater exercise time with RV pacing

**Table 2:**
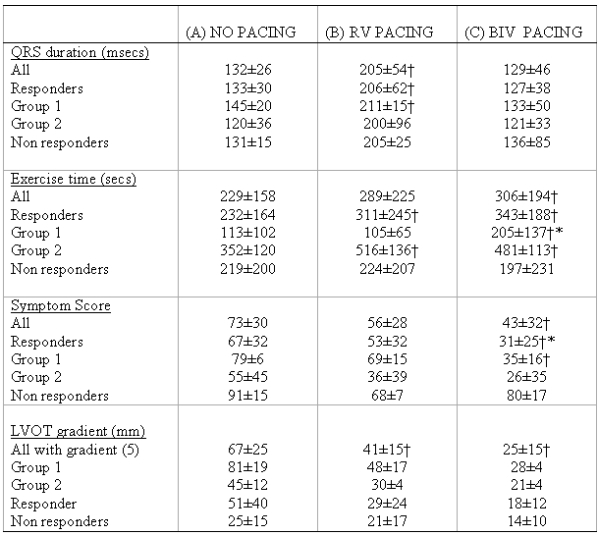
Endpoints: effects of pacing modalities on clinical, electrocardiographic and echocardiographic parameters

†P<0.05 vs no pacing, * P<0.05 vs no pacing and RV pacing

**Table 3:**
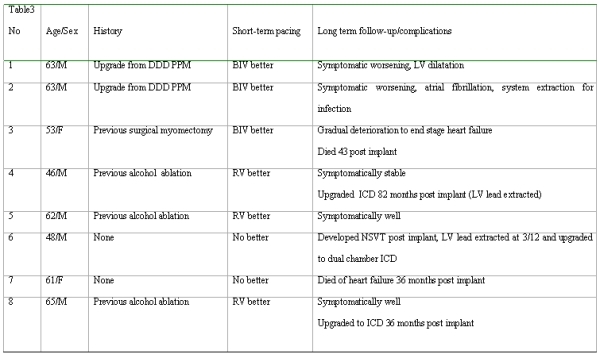
Long term follow of each patient
